# Improving vocational guidance through an expert system: Temporary decreasing and enhancing student self-awareness

**DOI:** 10.12688/f1000research.145109.1

**Published:** 2024-03-27

**Authors:** Julia Huayta-Gómez, Alex Pacheco

**Affiliations:** 1 Universidad Nacional de Cañete, San Vicente de Cañete District, Lima Region, Peru

**Keywords:** expert system, vocational guidance, Holland test, vocation

## Abstract

**Background:**

The vocational guidance process in educational institutions faces important challenges in managing trials and errors in diagnoses. Technological tools are identified as an effective solution to address these problems. This research seeks to improve career guidance in educational institutions through the implementation of an expert system. The main objective is to reduce test processing time and achieve greater efficiency in students’ self-knowledge regarding their interests, based on the personalities of the Holland Test.

**Methods:**

The development of the expert system followed a six-model approach. First, an organisational model was created to assess the scope and feasibility of the project. Next, a task and agent model was developed to investigate the impact and look for improvements. A knowledge model was then developed to analyse the relevant knowledge bases. A communication model was also developed to evaluate the communication interface of the system. Next, a design model was created to provide guidelines for the implementation of the system. Finally, the implementation of the knowledge system was carried out to ensure its correct functioning.

**Results:**

The implementation of the expert system has shown significant improvements in the vocational guidance process. It was possible to reduce the time needed to apply the test, thus optimising the psychologist’s time and allowing a greater capacity for analysis. In addition, an improvement in the effectiveness of the students’ self-knowledge in relation to their vocational interests based on the personalities of the Holland Test was observed.

**Conclusions:**

This study contributes to career guidance in educational institutions by introducing an innovative expert system. This technological solution optimizes the career guidance process, benefiting psychologists administering tests and students seeking self-knowledge about their career interests.

## Introduction

Expert systems were developed during the early 1960s and 1970s. An important quality they possess is that they provide fast and accurate problem solving and are able to solve a given problem and determine whether the problem is within their ability to solve. Their use is aimed at any field that requires human expertise; problems, in fact, become a potential scenario for a successful use of expert systems ( Laperrière & Reinhart, 2014; Kumar et al., 2023; Castilla et al., 2023). On the other hand, the structure of an expert system is organised around three main elements: Knowledge base, which consists of a large amount of information about a particular topic in a data structure on which the application is developed. Fact base, which is a working memory with data that remains unchanged about the situation in which the application is to be performed and the results that are obtained throughout the deduction process. Inference engine, is the core of the Expert System and implements the elements of the knowledge base, thus building reasoning and detecting the knowledge of interest, using and chaining them, building a solution plan without depending on the domain and specificity of the case treated ( Berrío & Ospina, 2014; Cabello, 2018). This helps solve problems that normally require human experts by mimicking the reasoning process that experts use to solve specific problems. In terms of vocational guidance, the expert system allows the student to identify his or her true interests and skills, as well as to have the necessary information about existing vocational options, while also being a support tool for the vocational counsellor ( Chatti et al., 2019; Castilla et al., 2020).

Vocational guidance is part of a very important process in the life of young people and adolescents because it favours the development of the future as a professional ( Vera et al., 2021; Quiroga et al., 2020). The process of career guidance is important because it is a form of psychological assistance aimed at helping counselees to elaborate their vocational identity and to be better able to make independent decisions to meet their own needs ( Miller, 2021; Admass, 2022). Career choice is an extension of the personality and an attempt to implement a particular style of behaviour in professional life. Therefore, vocational interest is basically just another aspect of personality and interest inventories (tests) ( Skarpaas & Hellekjær, 2021). People are divided into 6 different types, each corresponding to a work environment, which are: conventional, entrepreneurial, investigative, artistic, social and realistic ( Alfaro-Barquero & Chinchilla-Brenes, 2017; Van Thang et al., 2021). Each person projects their views about occupations, about themselves and about the work environment they prefer. This is done by using stereotypes, which are important in the psychological and sociological field.

It should be noted that in Ecuador ( Yungán et al., 2017), a research on an expert system to improve the assignment of teachers to different chairs achieved an efficiency of 93.33% compared to the manual, which had an efficiency of 33.33%.This indicates that an automated process outperforms the manual process by 60%, which is significantly improved by using the expert system. In Turkey ( Dahil et al., 2015) observed that in the current economic and market conditions, people need to continuously improve and renew their skills, so institutions should have implemented programmes that not only provide vocational and technical training, but also provide programmes to acquire broad and transferable skills, as well as occupation-specific skills, in order to meet the expectations for solving the problems of vocational and technical education in their country. In Spain ( Martínez-Vicente & Rocabert, 2016), it has been shown that vocational development is associated with career readiness. It can be concluded that, after career readiness, the students in the sample have sufficient career readiness and that career development plays an important role in achieving this. In Bolivia ( Alvarado-Gisbert, 2020), it was shown that it is crucial for students to be guided with vocational training, taking into account their professional profile, as well as having the necessary information about the academic offer, the work scenario, the demands and requirements of each career, which allows the student to plan their professional future. In Peru ( Flores & Gardi, 2020) it has been shown that the implementation of an expert system for the SGTI in a company manages to optimise the average time for the evaluation of the maturity levels, optimising the levels of reliability and efficiency. In Peru ( Barzola & Flores, 2017), the implementation of an Expert System for Vocational Guidance Support applied in an educational institution achieved great support in reducing the total time of the vocational guidance process, the total time of the analysis of the vocational test and increasing the total time of the interview with the student, fulfilling the purpose of supporting the work of the counsellor and the student.

At a global level, career guidance is very important because it allows for a better self-knowledge according to the needs of the environment and the changes experienced by society, specifically, it is a guidance process that is closely linked to the search for identity ( Bravo-Cobeña et al., 2021). It has the necessary tools to discover tastes, attitudes and skills in an objective and proactive way in the process of choosing a professional career. In Peru, the number of secondary school students applying to higher education institutions is really worrying; local statistics show that 4 out of 10 secondary school graduates apply to university ( INEI, 2015). In 2017, only 35.3% of secondary school graduates from the provinces of Lima enrolled in a higher education institution ( INEI, 2018). This indicates that there is a lack of career guidance and little use of technological tools for its application ( Casillas-González, 2021; Hasan et al., 2022).

The career guidance process faces a number of challenges that need to be better addressed in order to maximise its effectiveness and usefulness. Such as limited human resources and expertise, as the lack of trained psychologists or guidance counsellors in schools makes it difficult to provide personalised and specialised guidance. Diversity of interests and abilities, as students have a wide range of interests and abilities which can make it difficult to identify appropriate career options for each individual. Limited knowledge of options, as students may have limited knowledge of the different career options available in the country and schools often lack the capacity to provide up-to-date career information. Lack of tools and technology, the absence of technological tools and expert systems can hinder the efficient delivery of career guidance, lack of access to up-to-date information and interactive resources can limit the effectiveness of the process.

Vocational guidance is a process involving different actors and it is essential to establish fluid and collaborative communication in order to provide comprehensive support to students. The active participation of parents in this process is also essential, as they play a key role in supporting and guiding their children’s choices.

At present, many educational institutions carry out their career guidance processes manually, which leads to delays and errors due to the number of tests to be analysed, and in some cases career guidance is non-existent. This is particularly the case in the fifth year of secondary education in educational institutions such as I.E. P San Pedro - Quinocay, in the province of Yauyos, which carries out a simple process of vocational guidance that does not allow it to correctly orient its students and consequently less than 50% of the students are sure of the professional career they are going to follow ( Gutierrez-Valenzuela, 2021), This is due to various factors such as doubts and conflicts when choosing a career, difficulty in finding organised information about careers and higher education institutions, they do not manage to discover for themselves a professional field that is really attractive to them ( Martínez-Vicente & Rocabert, 2016). An expert system for career guidance facilitates the process of career guidance, which is done manually. Therefore, the objective of this research is to implement an expert system to reduce the time foreseen to carry out vocational guidance activities and the effectiveness of the student’s self-knowledge, guaranteeing a correct vocational guidance. This research describes the importance of an expert system for the improvement of the vocational guidance process, using as a reference a methodology applied exclusively in expert systems.

The innovation of this study lies in the use of technological tools and the development of an expert system to improve the process of career guidance in educational institutions. This approach addresses the problems of time constraints of the psychologist in charge, possible errors in diagnosis and delayed testing of students. Furthermore, the division of the expert system development into 6 models allows for a rigorous planning and design of the system, optimising the process of vocational guidance in a more effective and efficient way. The implementation of the knowledge system also improves students’ self-awareness, which is beneficial for both students and test administrators. In short, this innovation uses technology to improve and optimise the career guidance process in educational institutions.

## Methods

In this section, we provide a detailed description of the methods used in the development and operation of our expert system ( Huayta-Gómez & Pacheco, 2024).

### Application

Development technologies: Our expert system was meticulously crafted using a combination of cutting-edge technologies. The development of the Test rules and calculations is based on Python, a high-level, cross-platform, open-source programming language. The Front-end is based on Angular 11.0, an open source Javascript framework that guarantees a responsive and dynamic user interface, while the Back-end is based on C#, a modern object-oriented programming language. For the ApiRest services, use was made of Netcore 3.1, an open source cross-platform framework that aims to compile internet-connected and cloud-enabled applications. The database engine used was SQL Server 2019 for functional table maintenance data integrated with Netcore. The data model for solving Test Integrated with Python is based on MongoDB ( MongoDB Atlas, 2024).

Customisation for educational institutions: The basic framework of our software was customised to suit the specific requirements of educational institutions. For this purpose, the categorisation functions, user roles and results were adapted to the specific needs of the educational sector. The user roles are: Administrator, Tutor and Student.

### Operation

Minimum requirements of the expert system: Our expert system for the improvement of the vocational guidance process can operate on any web hosting platform that meets the following characteristics:

SQL Server 2019/MySQL

Windows 7 onwards

Server:

Intel Core™ i5 processor. RAM memory 8GB

Clients:

Intel Core™ i3 and above

### Unique features

This software tool has distinctive features that set it apart from existing solutions:

Personalised adaptation: The expert system is uniquely tailored to each student’s preferences, providing specific recommendations tailored to their profile.

Information: The expert system has information on higher education institutions, careers and professions of interest to the student.

Interactive: The expert system has an interactive environment that includes images and music, so that the student does not get bored in the process of answering the questions.

## Case studies

### Case study 1

**Student profile/vocational test**

**Figure 1.**
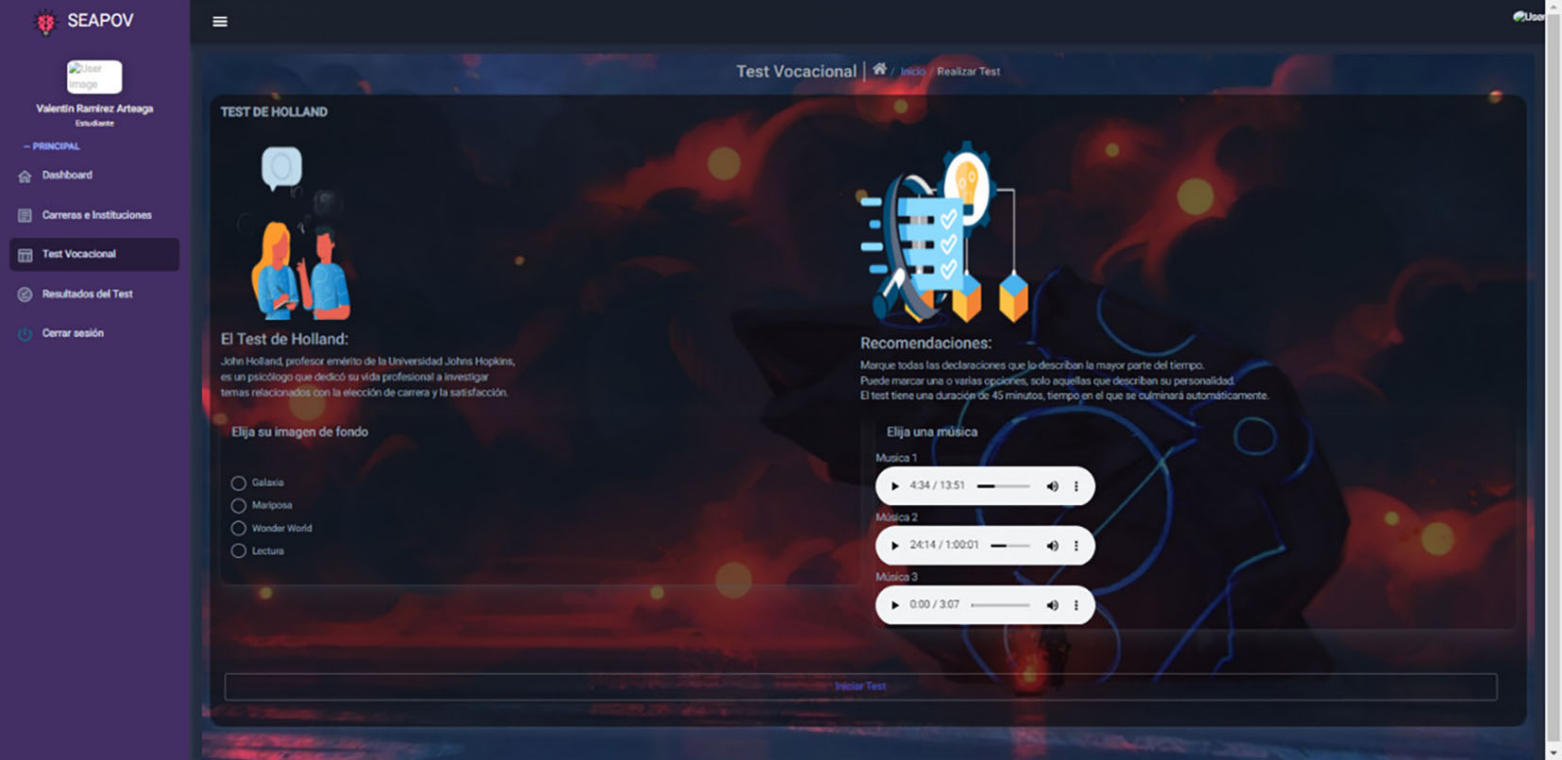
Beginning of vocational test. Source: self made.

**Figure 2.**
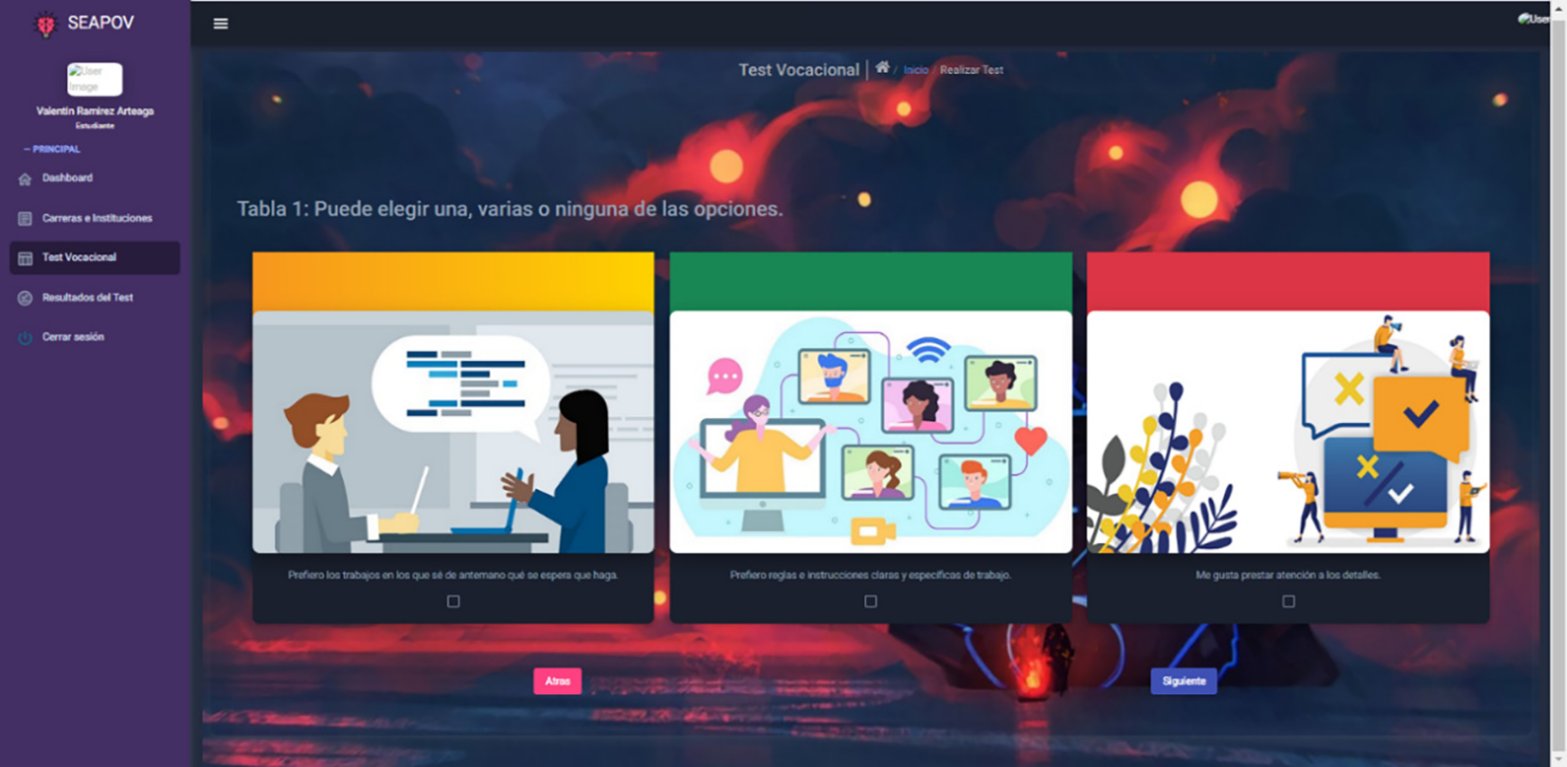
Vocational test questions. Source: self made.

**Figure 3.**
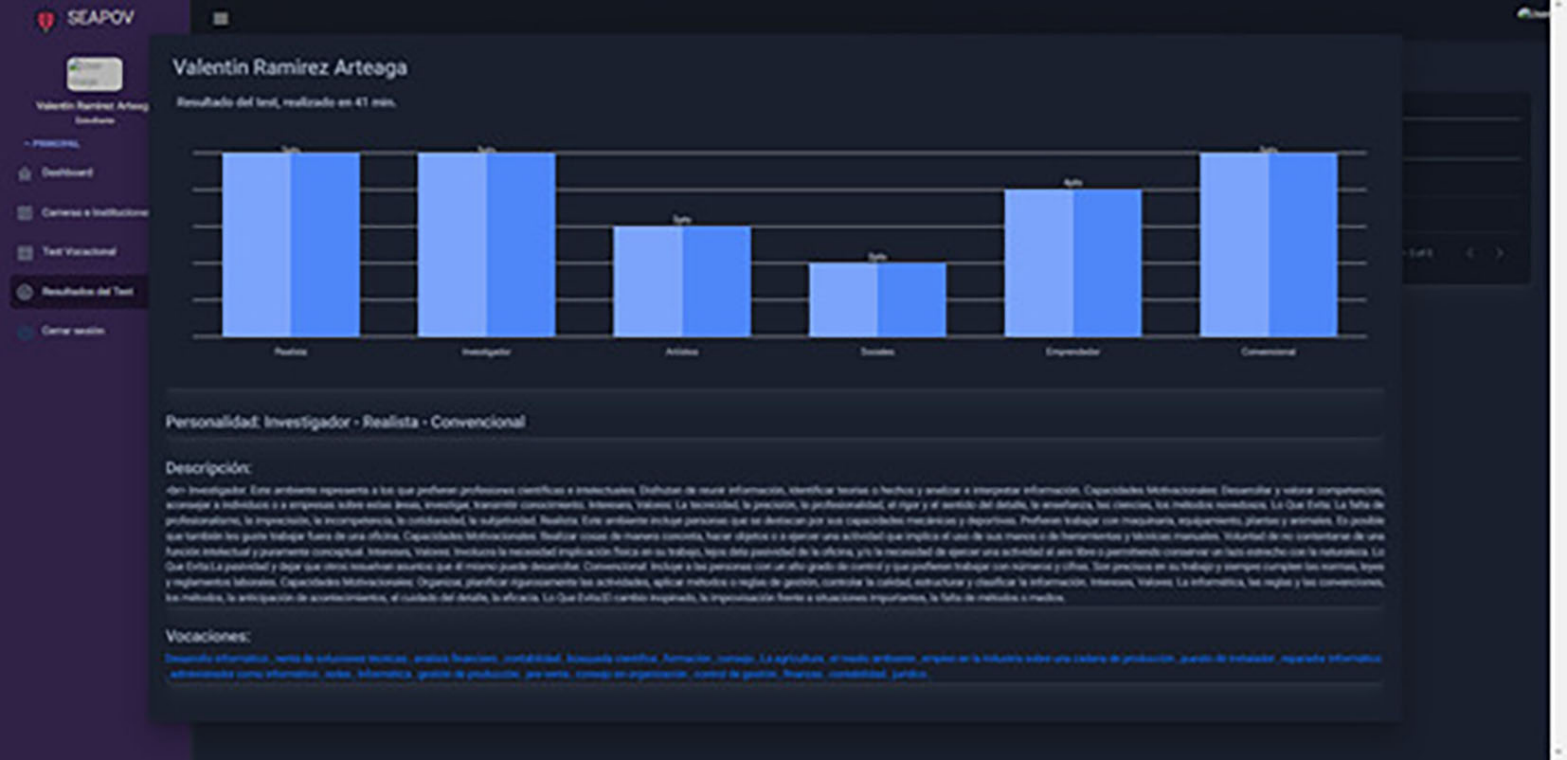
Vocational test results. Source: self made.

To demonstrate the functionality of the software, we present a specific use case involving the student taking the vocational test. In this case, the learner enters the Vocational Test section Figure 1, selects the background image he/she wishes to display during the test, such as music, in order to make it an interactive environment. Once the test has started, the student proceeds to answer the questions based on the Holland Test Figure 2, culminating with the completion of the test, the student can visualise his or her results and the recommendations of careers and vocations based on his or her profile Figure 3.

Input: Test answer input

Result: Holland personality test results and recommendations for vocations and careers according to your profile.

### Case study 2: Tutor profile/vocational tests taken by students

**Figure 4.**
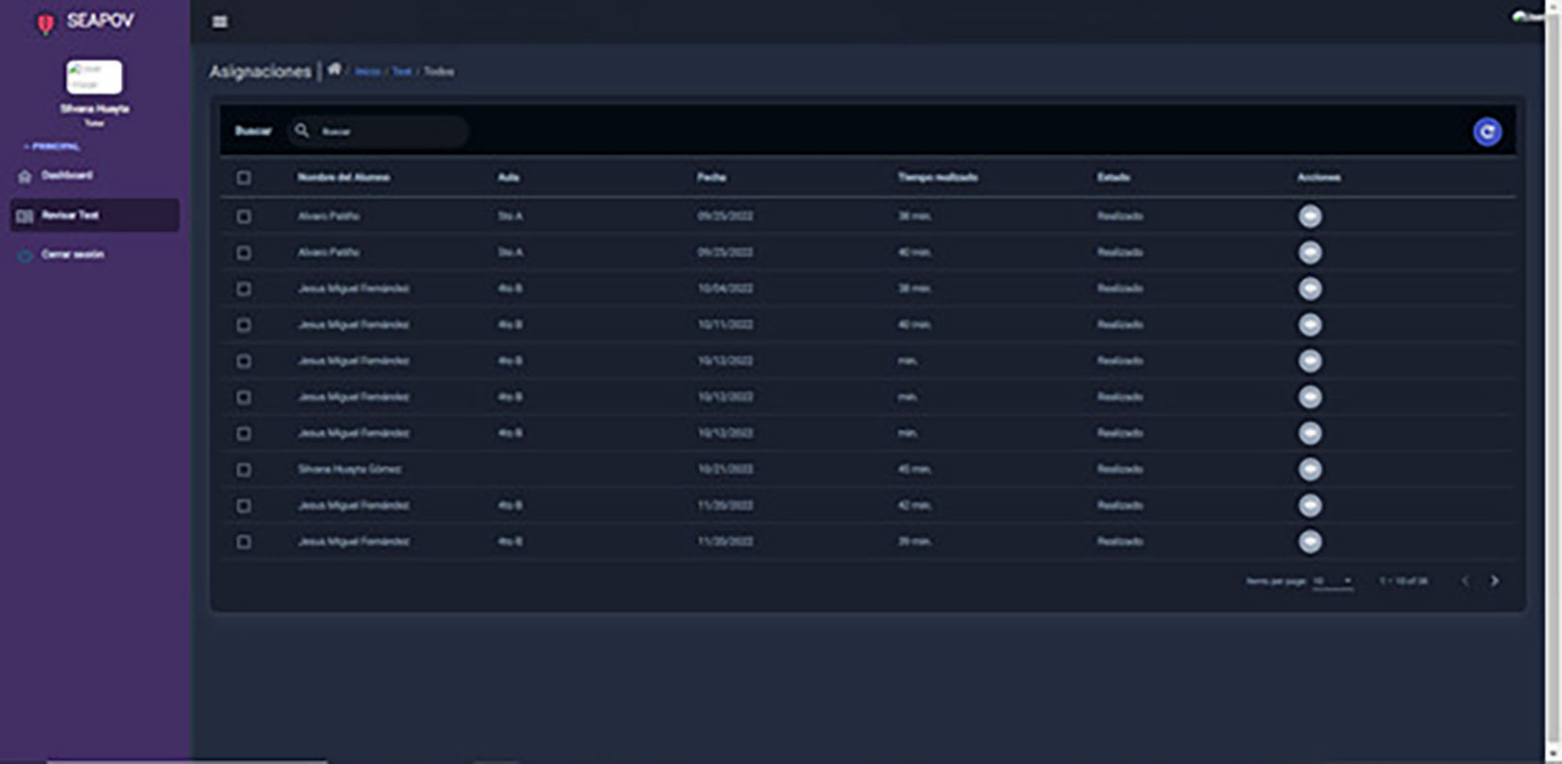
List of results of tests carried out by students under the care of a specific tutor. Source: self made.

**Figure 5.**
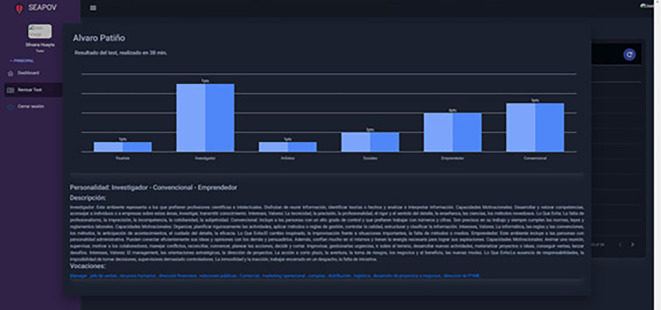
Viewing the results of the student in charge. Source: self made.

In this scenario, it becomes clear how the expert system makes it easier for the tutor or counsellor to visualise the results of the students in their care Figure 4, resulting in significant time savings by eliminating the need to assess each form test. This freed-up time can be effectively used to provide highly personalised guidance to each student, taking into account their particular interests and addressing any additional concerns related to the information provided by the expert system Figure 5.

Input:

Access the “Review Test” module.

Output:

Visualisation of the tests taken by the students in your charge.

Detail of results per student.

### Case study 3: Learner profile/Careers, professions and institutions

**Figure 6.**
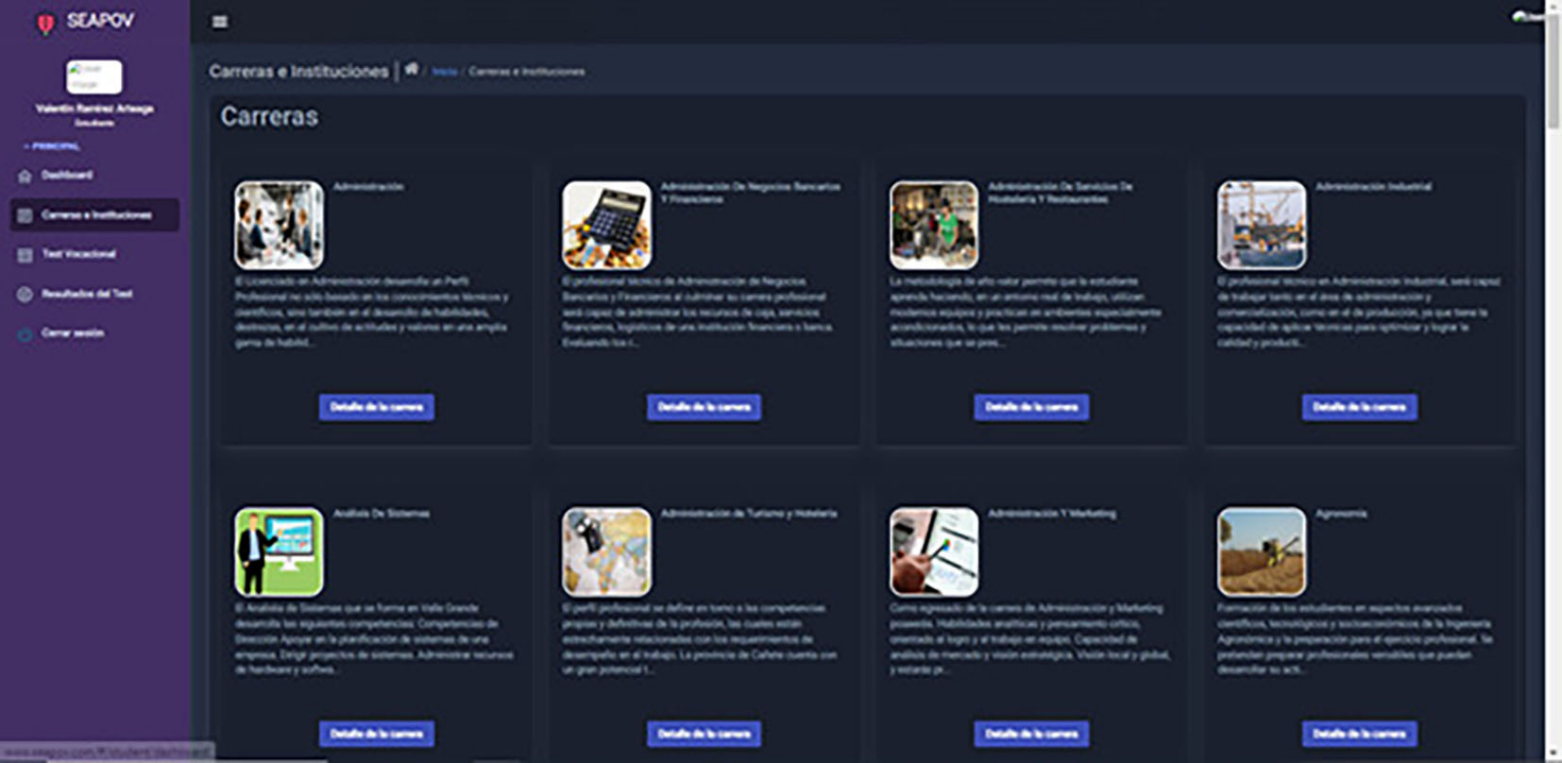
List of professional careers. Source: self made.

**Figure 7.**
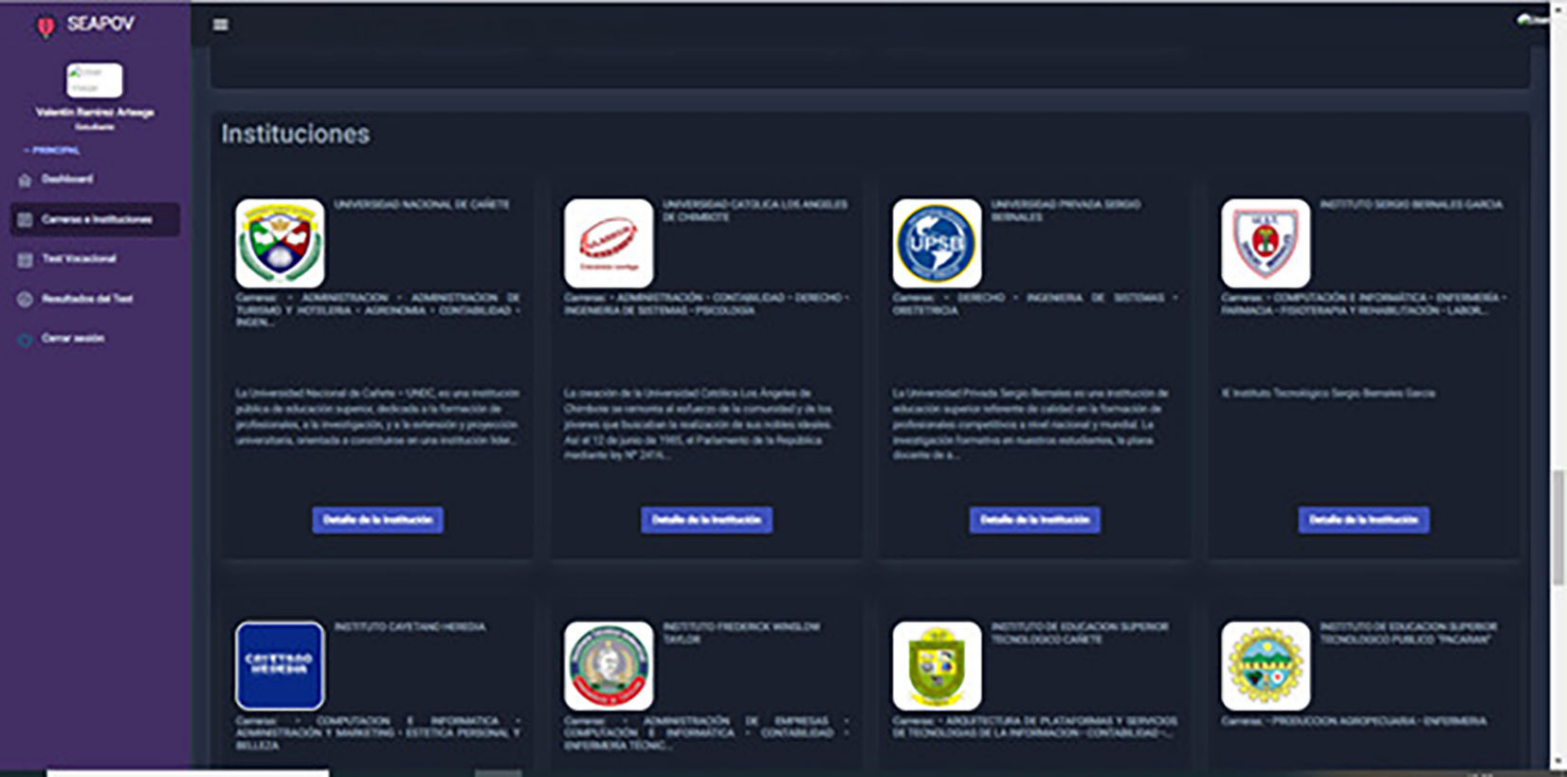
List of Higher Education Institutions. Source: self made.

**Figure 8.**
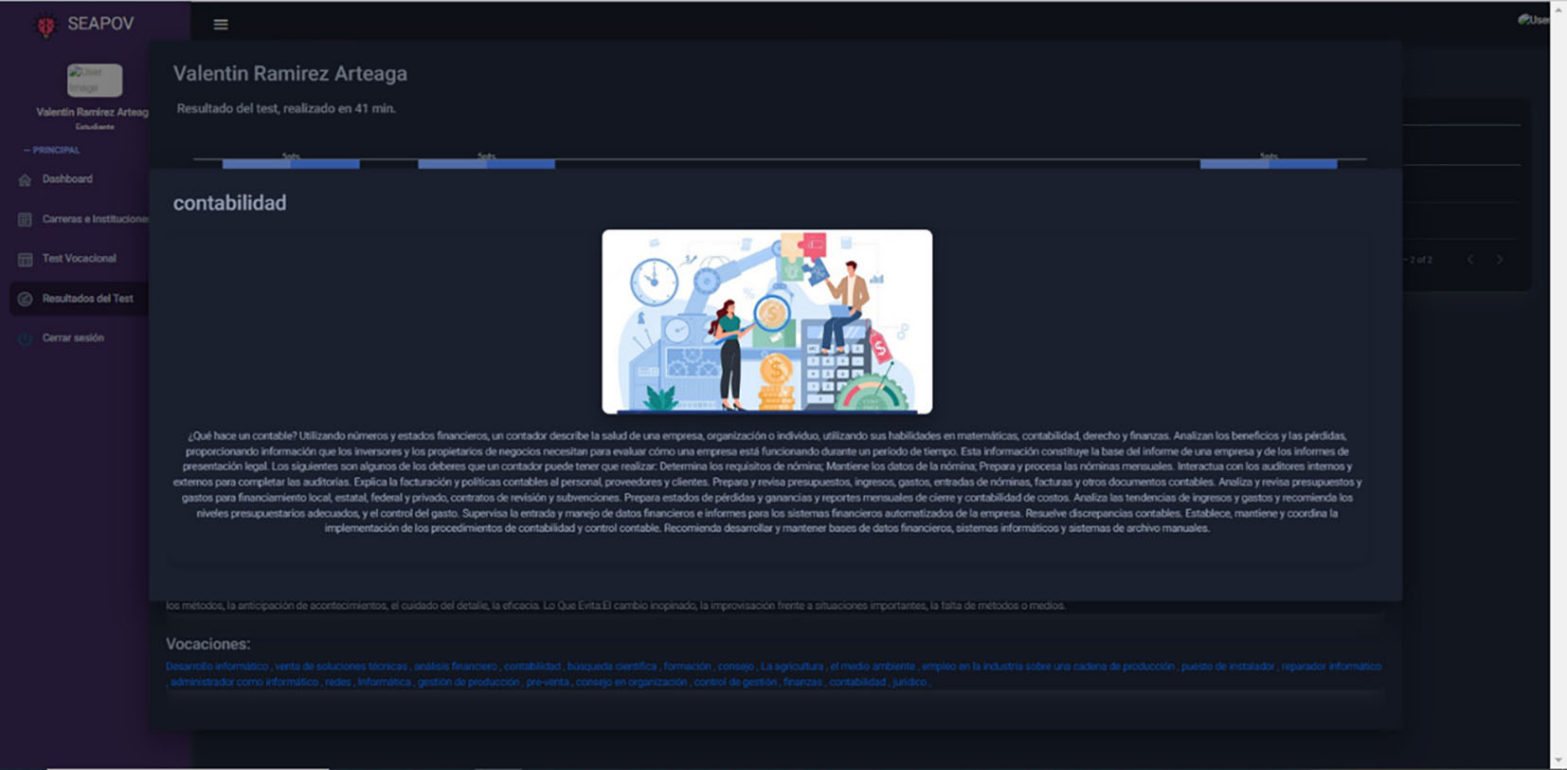
Additional information on careers and vocations. Source: self made.

In this scenario, the collection, processing and dissemination of relevant information about academic disciplines Figure 6, vocations Figure 8 and educational establishments Figure 7 takes place, enabling the student to acquire a more comprehensive knowledge about the opportunities available and the institutions where he/she could acquire his/her future training, as well as the responsibilities inherent to the practice of the vocation of his/her choice.

Input: Access the module “Careers and Institutions”.

Output: Information on careers, vocations and institutions.

These use cases demonstrate how the software enhances the vocational guidance process for students in an educational institution by providing valuable tools and necessary information.

## Discussion

The study highlights the ability of the expert system to adapt to the individual preferences and characteristics of the user, adding an important dimension of interaction and engagement. The immediate feedback provided in the form of results and recommendations can be critical to the student’s career decision-making process, clearly demonstrating how the software adds value to the career guidance process by combining technology, personalisation and accurate feedback. As a result, the software presents itself as a promising tool for improving the effectiveness and experience of careers guidance. These results show a significant difference before and after the implementation of the expert system in the improvement of the participants’ self-awareness, as well as an increased reliability of the results. This is in line with ( Farahani et al., 2023) who conclude that an expert system provides a systematic and structured framework for analysing data, allowing for more reliable and accurate diagnosis. These findings support the reliability of the results.

The study shows how the expert system becomes a valuable tool for the tutor as it facilitates access to student performance information without the need to spend time analysing test answers to obtain results, allowing the tutor to focus more on the individual needs of each student. These results support the previous claims of ( Barzola & Flores, 2017), who showed that the implementation of an expert system can reduce the expected time to complete activities, reducing the time of the tutoring process by approximately 7.65 hours. In addition, the findings are also consistent with those of ( Orbezo, 2017) who obtained an average reduction in analysis time of 20.3%. It is also in line with the results of ( Castillo, 2013) who concluded that career guidance can be simplified and improved through an expert system, which has a positive impact on the efficiency of the process, especially for young people.

The study demonstrates the ability of the expert system to facilitate easier and faster access to key information. By collecting and processing data on academic disciplines, professions and higher education institutions based on the student’s profile, the system creates a space where students can explore a wider range of career options in a more informed way. The dissemination of this information is crucial to enable students to make informed decisions about their academic and professional future. These findings support the conclusions of ( Chacon, 2015), who postulates that the implementation of an expert system broadens the scope of career guidance by enabling more students to access appropriate guidance for their future careers. This is achieved by providing essential information about the different career options available, as well as a test that allows students to accurately identify their true interests and abilities.

## Conclusions

The use of expert systems in the professional process has proved to be an effective solution to meet the challenges and improve the quality of this process in schools. The following conclusions can be drawn from the research carried out

1. Reduction of the time needed: Expert systems make it possible to streamline the careers guidance process by reducing the time needed to administer tests and carry out assessments. This optimises the time of guidance psychologists, allowing them to serve a larger number of students and to analyse the results more thoroughly.
2. Improving the effectiveness of self-awareness: By using expert systems such as the Holland Test, students can learn more about their interests and vocations. These systems analyse students’ answers and provide information on career options that match their characteristics. This provides more accurate and personalised guidance.
3. Increasing the efficiency of the decision-making process: Expert systems provide students with objective and proactive information for career choice. By providing appropriate tools and resources, they help students make informed choices and develop independent decision-making skills.
4. Optimisation of the career guidance process: The implementation of expert systems in educational institutions benefits both psychologists and students. These systems enable more efficient administration of vocational tests, improve the quality of diagnoses and provide additional support to guidance counsellors.

### Ethical considerations and consent of participants

The study was carried out following the ethical principles approved by the Ethics Committee of the National University of Cañete on August 1, 2022, reference number: std003-010822. Furthermore, the guidelines of the Declaration of Helsinki were also followed throughout the study. Respondents gave written consent to conduct the investigation and process data. Written consent was provided prior to the survey. Respondents could not continue with the survey if they were unwilling to consent or otherwise withdraw.

## Data Availability

Zenodo: Data from the expert system for the vocational guidance process, https://doi.org/10.5281/zenodo.10794555 ( Pacheco & Huayta-Gómez, 2024). This project contains the following underlying data:
- Pretest and Posttest.xlsx The data are available under the terms of the Creative Commons Attribution 4.0 International (CC-BY 4.0) licence.

## Software availability

Huayta, J. & Pacheco, A. Improving Vocational Guidance through an Expert System: Temporary Decreasing and Enhancing Student Self-awareness

• Source code available from: https://github.com/Inefable11/HollandTestProject/releases/ ( Huayta-Gómez & Pacheco, 2024).
• Archived source code at time of publication: https://doi.org/10.5281/zenodo.10712729 ( Huayta-Gomez & Pacheco 2024).

The software are available under the terms of the Creative Commons Attribution 4.0 International (CC-BY 4.0) licence
