# Supplementary material for: Improving vocational guidance through an expert system: Temporary decreasing and enhancing student self-awareness
Source: F1000Res. 2024 Mar 27;13:232. [Version 1] doi: 10.12688/f1000research.145109.1 (PMC11187528; doi:10.12688/f1000research.145109.1)
Supplement: Peer review history

# Reviewer report: Reviewer response for version 1

**DOI:** 10.5256/f1000research.159001.r283550

**Published:** 2024-06-19

**Authors:** Eko Pramudya Laksana

**Affiliations:** 1 Universitas Negeri Malang, Malang, East Java, Indonesia

In introduction, it would be advantageous to incorporate research concerning the counseling and guidance process that involves the utilization of established instruments.

 This article significantly advances our understanding of expert systems, presenting findings that are highly relevant and carry important implications. However, the data analysis requires further depth, necessitating the incorporation of more robust arguments.

 The author effectively integrates the research findings with pertinent theoretical frameworks, thereby establishing a robust foundation for the results obtained. This article offers a comprehensive and insightful examination of the topic, demonstrating significant potential as a basis for future scholarly investigation.

Is the work clearly and accurately presented and does it cite the current literature?

Yes

If applicable, is the statistical analysis and its interpretation appropriate?

Yes

Are all the source data underlying the results available to ensure full reproducibility?

Yes

Is the study design appropriate and is the work technically sound?

Yes

Are the conclusions drawn adequately supported by the results?

Yes

Are sufficient details of methods and analysis provided to allow replication by others?

Yes

Reviewer Expertise:

School counseling; guidance and counseling; education

I confirm that I have read this submission and believe that I have an appropriate level of expertise to confirm that it is of an acceptable scientific standard.

# Reviewer report: Reviewer response for version 1

**DOI:** 10.5256/f1000research.159001.r274749

**Published:** 2024-06-07

**Authors:** Sri Tutur Martaningsih

**Affiliations:** 1 Ahmad Dahlan University, Yogyakarta, Special Region of Yogyakarta, Indonesia

Having novelty and good benefits, it is a starting point that can be developed to be used more widely. Moreover, by integrating it with technological developments, it enables easy access and use.

 In my opinion, career directions are developing increasingly diverse and more specific. On the other hand, disruption occurs in various fields of work. This makes demands for individual competence and readiness to be relevant to new areas of work. Therefore, the expert system used needs to be developed with increasingly specific substance. Vocational schools have many departments, each with relevant competency and character prerequisites. For this reason, detecting  suitability of competencies and talents. As well as: Interests and characters needs to be made more clear. It is hoped that the expert system can fulfill the need for individual career planning to be more effective.

 Test products on a wider range of subjects, if necessary across countries with different cultures and individual characteristics. A wider range of test subjects will enrich the feedback obtained, in order to continuously improve the expert system being developed.

Is the work clearly and accurately presented and does it cite the current literature?

Yes

If applicable, is the statistical analysis and its interpretation appropriate?

Partly

Are all the source data underlying the results available to ensure full reproducibility?

Partly

Is the study design appropriate and is the work technically sound?

Yes

Are the conclusions drawn adequately supported by the results?

Yes

Are sufficient details of methods and analysis provided to allow replication by others?

Yes

Reviewer Expertise:

Educational research and evaluation

I confirm that I have read this submission and believe that I have an appropriate level of expertise to confirm that it is of an acceptable scientific standard.
